# Supplementary material for: Metal-Responsive Transcription Factors Co-Regulate Anti-Sigma Factor (Rsd) and Ribosome Dimerization Factor Expression
Source: Int J Mol Sci. 2023 Mar 1;24(5):4717. doi: 10.3390/ijms24054717 (PMC10003395; doi:10.3390/ijms24054717)
Supplement: Supplementary file 1 [file ijms-24-04717-s001.zip › ijms-2155101-supplementary.pdf]

International Journal of
Molecular Sciences

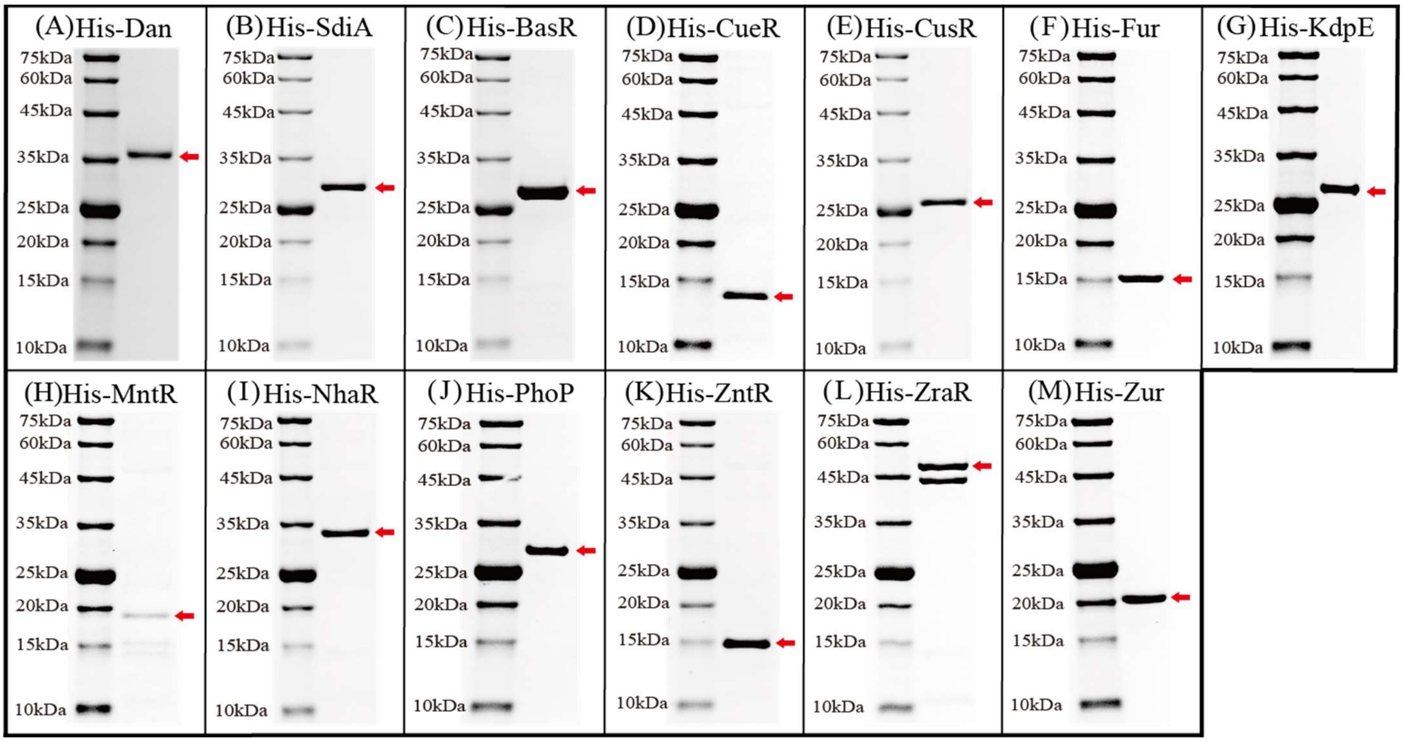

Figure S1. Analysis of purified transcription factors using SDS-PAGE. His-tagged transcription factors used in the in vitro assay were purified through columns, and expression was confirmed through SDS-PAGE. Target bands are indicated using red arrows.

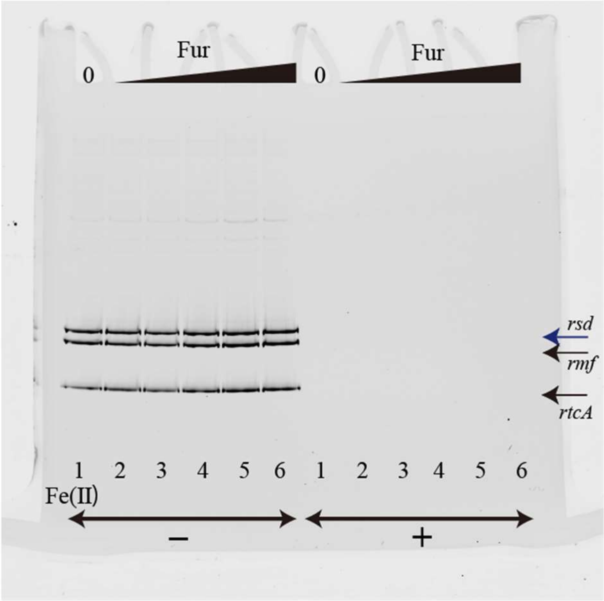

Figure S2. Influence of effector ($FeSO_4$) on the DNA-binding activity of Fur. A mixture of 0.5 pmol each of fluorescein-4-isothiocyanate-labeled *rsd*, *rmf*, and *rtcA* probes was incubated with increasing concentrations of transcription factors (lanes 1 to 6: 0, 0.5, 1, 2, 4, and 8 pmol, respectively) in the presence of 10 mM effector ($FeSO_4$).

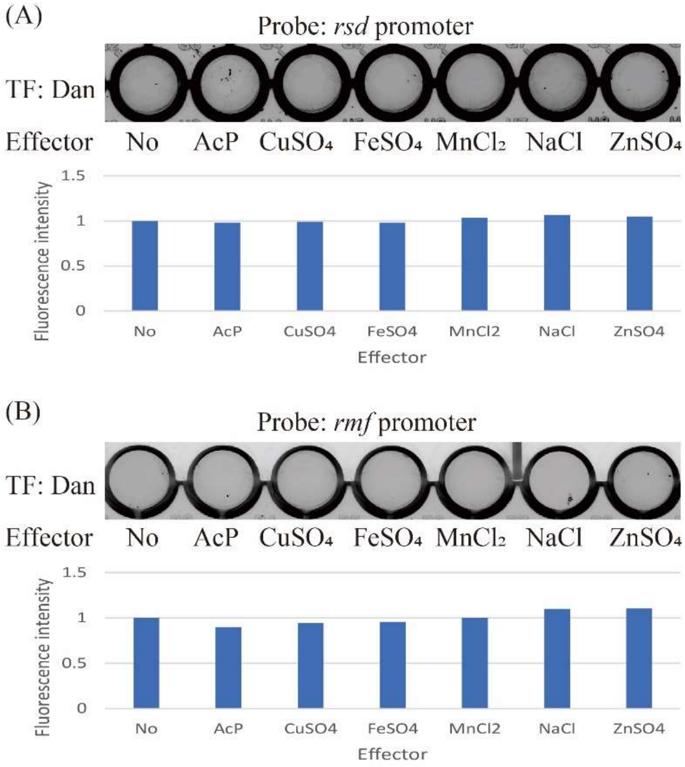

Figure S3. Beads assay with effector added to Dan (His-tagged protein) and the *rsd* (A)and *rmf* (B) promoter. Each upper panel: A scanned image. Each lower panel: The relative intensity of fluorescence for effector addition, which is normalized against the value of no effector. The amounts of probes, Dan, and effectors added are described in Section 4.5.

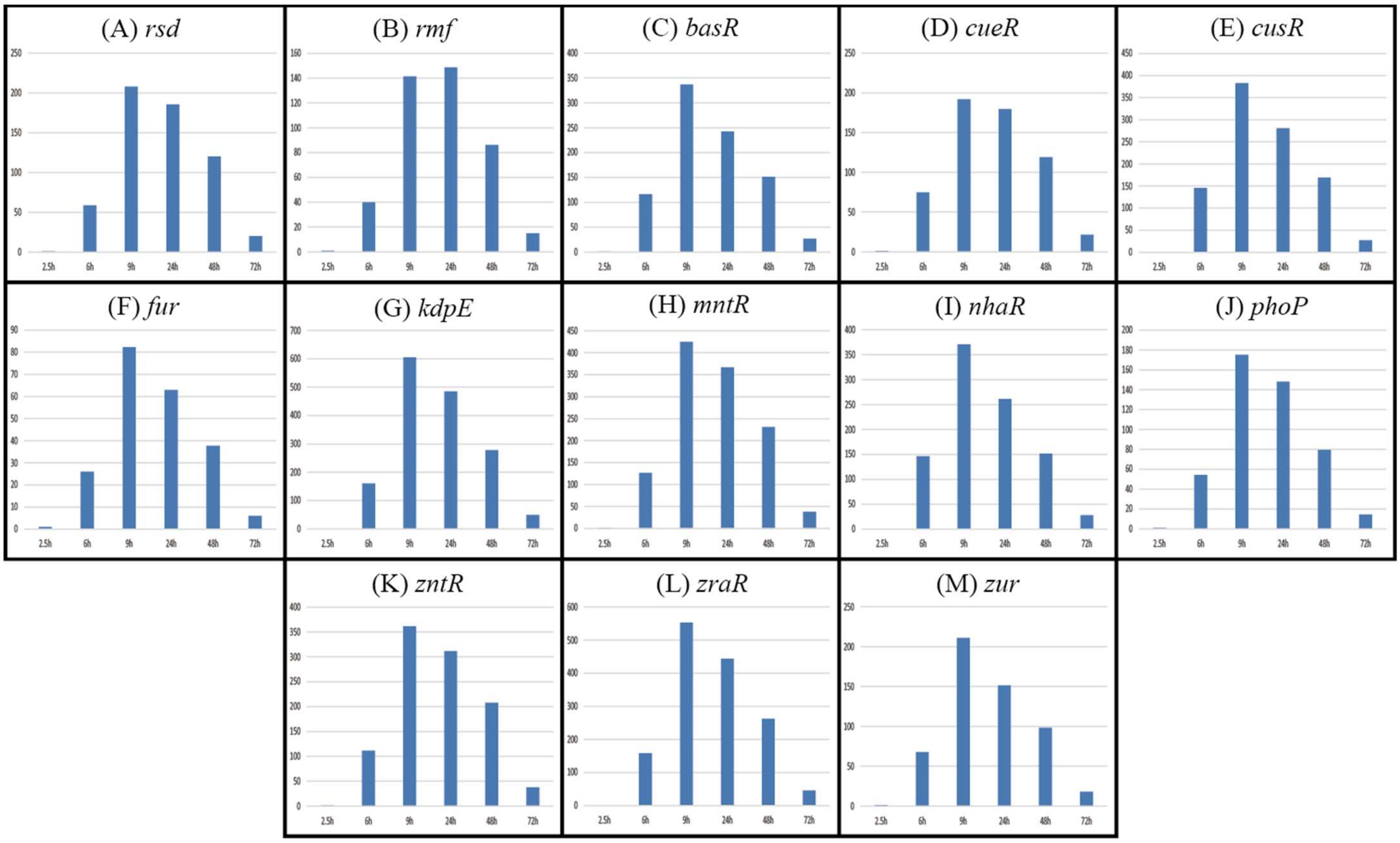

Figure S4. Amount of mRNA transcribed from each target gene. After the beginning of incubation, the parental cell was cultured under aerobic conditions for up to 72 h in medium E (containing 2% polypeptone and 0.5% glucose) at 37°C with 160 rpm shaking. The cells were harvested at 2.5, 6, 9, 24, 48, and 72 h, and the amount of target mRNA was measured through qPCR. The values were normalized at 2.5 h.
